# Construction of a Prognostic Immune-Related LncRNA Risk Model for Lung Adenocarcinoma

**DOI:** 10.3389/fcell.2021.648806

**Published:** 2021-03-18

**Authors:** Yue Li, Ruoyi Shen, Anqi Wang, Jian Zhao, Jieqi Zhou, Weijie Zhang, Ruochen Zhang, Jianjie Zhu, Zeyi Liu, Jian-an Huang

**Affiliations:** ^1^Department of Pulmonary and Critical Care Medicine, The First Affiliated Hospital of Soochow University, Suzhou, China; ^2^Suzhou Key Laboratory for Respiratory Diseases, Suzhou, China; ^3^Institute of Respiratory Diseases, Soochow University, Suzhou, China

**Keywords:** lung adenocarcinoma, long noncoding RNA, risk model, immune check point, biology message

## Abstract

**Background:**

Lung adenocarcinoma (LUAD) originates mainly from the mucous epithelium and glandular epithelium of the bronchi. It is the most common pathologic subtype of non-small cell lung cancer (NSCLC). At present, there is still a lack of clear criteria to predict the efficacy of immunotherapy. The 5-year survival rate for LUAD patients remains low.

**Methods:**

All data were downloaded from The Cancer Genome Atlas (TCGA) database. We used Gene Set Enrichment Analysis (GSEA) database to obtain immune-related mRNAs. Immune-related lncRNAs were acquired by using the correlation test of the immune-related genes with R version 3.6.3 (Pearson correlation coefficient cor = 0.5, *P* < 0.05). The TCGA-LUAD dataset was divided into the testing set and the training set randomly. Based on the training set to perform univariate and multivariate Cox regression analyses, we screened prognostic immune-related lncRNAs and given a risk score to each sample. Samples were divided into the high-risk group and the low-risk group according to the median risk score. By the combination of Kaplan–Meier (KM) survival curve, the receiver operating characteristic (ROC) (AUC) curve, the independent risk factor analysis, and the clinical data of the samples, we assessed the accuracy of the risk model. Gene Ontology (GO) enrichment analysis and Kyoto Encyclopedia of Genes and Genomes (KEGG) enrichment analysis were performed on the differentially expressed mRNAs between the high-risk group and the low-risk group. The differentially expressed genes related to immune response between two risk groups were analyzed to evaluate the role of the model in predicting the efficacy and effects of immunotherapy. In order to explain the internal mechanism of the risk model in predicting the efficacy of immunotherapy, we analyzed the differentially expressed genes related to epithelial-mesenchymal transition (EMT) between two risk groups. We extracted RNA from normal bronchial epithelial cell and LUAD cells and verified the expression level of lncRNAs in the risk model by a quantitative real-time polymerase chain reaction (qRT-PCR) test. We compared our risk model with other published prognostic signatures with data from an independent cohort. We transfected LUAD cell with siRNA-LINC0253. Western blot analysis was performed to observed change of EMT-related marker in protein level.

**Results:**

Through univariate Cox regression analysis, 24 immune-related lncRNAs were found to be strongly associated with the survival of the TCGA-LUAD dataset. Utilizing multivariate Cox regression analysis, 10 lncRNAs were selected to establish the risk model. The K-M survival curves and the ROC (AUC) curves proved that the risk model has a fine predictive effect. The GO enrichment analysis indicated that the effect of the differentially expressed genes between high-risk and low-risk groups is mainly involved in immune response and intercellular interaction. The KEGG enrichment analysis indicated that the differentially expressed genes between high-risk and low-risk groups are mainly involved in endocytosis and the MAPK signaling pathway. The expression of genes related to the efficacy of immunotherapy was significantly different between the two groups. A qRT-PCR test verified the expression level of lncRNAs in LUAD cells in the risk model. The AUC of ROC of 5 years in the independent validation dataset showed that this model had superior accuracy. Western blot analysis verified the change of EMT-related marker in protein level.

**Conclusion:**

The immune lncRNA risk model established by us could better predict the prognosis of patients with LUAD.

## Introduction

At present, the incidence of malignant tumors is rising gradually, posing a serious threat to human health. With the continuous development of medical molecular biology, tumor immunology, tumor genetics, and biological engineering, the mechanism of the occurrence and development of malignant tumors has been explored step by step. The related proteins and driver genes of malignant tumors have been gradually discovered, providing more and more targets for molecular targeted therapy and immunotherapy of tumors. Nowadays, molecular targeted therapy and immunotherapy have become new and important therapeutic methods after traditional surgery, radiotherapy, and chemotherapy (Herbst et al., 2018; Jemal et al., 2018). Lung cancer is one of the most common malignant tumors, more than 80% of are NSCLC. The incidence and mortality of lung cancer top the list of all kinds of malignancies. However, lung cancer is frequently diagnosed in advanced stage because of the lacking of early specific symptoms, posing a serious public health burden. Due to the low rate of early diagnosis, only about 20% of patients are surgically removed at an early stage. In addition, in which 5% have a distant recurrence after surgery. The 5-year survival rate for lung cancer is only about 10%. In recent years, molecular targeted therapy and immunotherapy have become the most popular and promising research in the fields of NSCLC. Nevertheless, such patients have to bear high costs in most cases, and most of them will develop drug resistance within one to 2 years. Therefore, it is important to actively explore the pathogenesis of NSCLC and seek new directions for diagnosis and treatment (Soria et al., 2018). LncRNA is a gene therapy target that is closely related to the occurrence and development of tumors. LncRNA is a kind of RNA whose length is larger than 200 bases and lacks the ability of protein-coding. A large number of studies have shown that many lncRNAs play a pivotal role in tumor cell activity. LncRNAs are involved in multi-gene regulatory networks and can be used as biomarkers for early tumor detection and prognosis (van Leeuwen and Mikkers, 2010). LncRNAs can be divided into two basic types according to their comparative characteristics with mRNA: intergenic and intronic. It can also be classified according to the direction of lncRNA and mRNA: bidirectional, sense, antisense. LncRNAs contain promoter-associated sequences, but no open reading frame (ORF). Their main function is to regulate the expression of target genes at many levels. LncRNAs can be complementary with parts of the mRNAs sequences to regulate protein expression and maintain the balance of functional proteins and signaling pathways in cells. With the discoveries of new lncRNAs and the clear studies of relevant mechanisms, lncRNAs are expected to bring new changes to the basic and clinical of NSCLC. The TCGA database includes gene expression, protein expression, DNA methylation, gene copy number, providing a platform for searching cancer-specific characteristics (Xu et al., 2015). Although the TCGA database could predict prognostic biomarkers of cancers, it is still a classic problem that whether molecular biomarkers could forecast the prognosis of patients with LUAD. In this study, information downloaded from the TCGA dataset was comprehensively analyzed. The prognostic value of key immune-related lncRNAs was evaluated in combination with the clinical characteristics of the patients.

In this study, we identified the expression of immune-related lncRNAs in patients with LUAD. Cox regression model was used to identify an immune-related lncRNAs signature that was used to construct prognostic model. We identified an immune-related 10-lncRNA signature associated with the prognosis of LUAD patients in the training set that performed well in the testing set and the TCGA dataset. Finally, we further verified that these 10 lncRNAs were significantly differentially expressed between the human normal bronchial epithelium cell line BEAS2B and lung adenocarcinoma (LUAD) cell lines (A549, H1299) by a qRT-PCR test. We transfected LUAD cell with siRNA-LINC0253. Western blot analysis was performed to observe the change of EMT-related marker in protein level. When knockdown LINC02535, we observed SNAIL, MMP2, and N-Cadherin (CDH2) decrease.

This immune-related 10-lncRNAs signature not only can improve the ability to predict prognosis in patients with LUAD but also can promote better clinical strategies and elucidate the underlying mechanisms.

## Materials and Methods

### Acquisition of TCGA Data

All data of the LUAD patients were downloaded from the TCGA database^1^.

### Acquisition of the Immune Genes

The list of immune genes was obtained through the GSEA database^2^.

### Screening Immune-Related lncRNAs

We screened the differentially expressed lncRNAs (fold change = 1.0, *P* < 0.05). Immune-related lncRNAs were obtained by using the correlation test of the immune genes with R version 3.6.3 (Pearson correlation coefficient cor = 0.5, *P* < 0.05).

### Construction of Immune-Related lncRNAs Risk Score Model

To get a prognostic model, We downloaded the survival time and survival status of the patients from the TCGA database (see text footnote 1). The TCGA-LUAD dataset was randomly divided into the testing set and the training set by R software. Based on the training set, univariate Cox regression analysis and multivariate regression Cox analysis were performed to construct immune-related lncRNAs risk score model by using the R package “glmnet.” The risk score for each patient was as follows:

$$\mathrm{Risk}\,\mathrm{score}=\sum_{i=1}^{n}\beta_{i}\,\chi_{i}$$

The β_*i*_ represents the multivariate regression Cox analysis coefficient of each gene. The χ_*i*_ represents the expression of each lncRNA. The accuracy of the risk model was assessed by using the Kaplan–Meier (KM) curve and the ROC (AUC) curve.

### Independent Prognostic Factor Analysis

The information about 309 patients with complete clinical data on TCGA-LUAD was combined with the risk score, and the independent prognostic factor was analyzed by using the “survival” package of R software.

### GO Enrichment Analysis and KEGG Enrichment Analysis for the Differentially Expressed Genes Between the High-Risk Group and the Low-Risk Group

Using the “clusterProfiler” package of R software to conduct GO enrichment analysis and KEGG enrichment analysis.

### Analyzing Differentially Expressed Genes Related to the Immune Response Between the High-Risk Group and the Low-Risk Group

Using the “limma” package and the “ggpubr” package of R software to analyze differentially expressed genes related to immune response between the high-risk group and the low-risk group.

### Analyzing Differentially Expressed Genes Related to EMT Between the High-Risk Group and the Low-Risk Group

Using the “limma” package and the “ggpubr” package of R software to analyze differentially expressed genes related to EMT between the high-risk group and the low-risk group.

### Cell Culture

The human normal bronchial epithelium cell line BEAS2B and LUAD cell lines A549, H1299 were purchased from the Cell Bank of the Chinese Academy of Sciences (Shanghai, China). All cell lines were cultured in RPMI-1640 medium (Gibco, China), respectively. All media were supplemented with 10% fetal bovine serum (Gibco, China). Cells were grown at 37°C in an atmosphere of 5% CO_2_ and were tested without mycoplasma.

### RNA Extraction and Quantitative Real-Time PCR

Total RNA was extracted from cells with RNAiso Plus (Takala, Japan). Then, 1 μg of total RNA was reverse-transcribed with a NanoDrop2000 (Thermo Fisher Scientific, United States) in a 20-μL reaction according to the manufacturer instructions. Quantitative real-time PCR was performed with SYBR QPCR Mix (Takala, Japan) in a 20-μL reaction containing 1 μL of cDNA and was run on an ABI Step One Plus Real-Time PCR system (Applied Biosystems). The PCR primer sequences directly were synthesized (Sangon Biotech, China) (Table 1). After being briefly mixed, the reaction mixture was at 95°C for 10 min, followed by 40 cycles at 95°C for 15 s and 60°C for 1 min. β-Actin was used as an endogenous control to standardize the expression of each target gene, and the 2^–ΔΔCT^ method was adopted to determine the relative target gene level. Statistical analyses were performed with GraphPad Prism.

**TABLE 1:** qRT-PCR primer sequences.

LncRNA	Sequence
LINC02535	Forward: 5′-GGCTGGTTGTGGTGGCTCATG-3′
	Reverse: 5′-TTGCGATATTGCCCAGGCTTGTC-3′;
AL034397.3	Forward: 5′-AGGCACCACTCCACTGACAGAC-3′
	Reverse: 5′-CCCTGGCAAAGTTGTTGGAAAGTG-3′;
AC007639.1	Forward: 5′-GCTGACTCGGTGGGTGCTTTG-3′
	Reverse: 5′-GAGGCTGAGGTGGGAGGATCG-3′;
CHODL-AS1	Forward: 5′-AGCACTCAGCACCAGCACAAAC-3′
	Reverse: 5′-GCAGGTCAGCTTCAGTTGGAGATC-3′;
AL078645.1	Forward: 5′-GCAGGTATTGTCAGTAGGGCAAGG-3′
	Reverse: 5′-TCCCAAGCATGGAAACAGGTTCAC-3′;
LINC01878	Forward: 5′-TGTGGGAAGCAGGTTCAGGATTTC-3′
	Reverse: 5′-TGCCACTTTCCCAATCACGAAGAG-3′;
AL031600.2	Forward: 5′-CCAGCAAGGAATAGCCTGAGAAGC-3′
	Reverse: 5′-GGACACACCCTGCCCAGAGG-3′;
AC090518.1	Forward: 5′-TGTTGCCCTGTTCACCGAAATCC-3′
	Reverse: 5′-TTTCCTTGCCTGTTGTCCTCTGTG-3′;
LINC02412	Forward: 5′-CTGGAGCAGGAGCCTCAGTCTC-3′
	Reverse: 5′-TCTGGTGTCTGGAAGGGATGATGG-3′;
AC018607.1	Forward: 5′-TGATCCTCCTGCCTCAGCTTCTC-3′
	Reverse: 5′-TCCAGTGCCTGTGCATGTTCTTC-3′

### RNA Interference

Two pre-designed small interfering RNA (siRNA) sequences targeting different regions of LINC02535 were directly synthesized (GenePharma). The target sequences of the siRNAs were as follows: siRNA-LINC02535–1: 5′-GCC GAT TGC TCA CAA AGA T-3′; siRNA-LINC02535–2: 5′-GCA TAC AAT GGG ACA GTT T-3′. Scrambled siRNA was used as a negative control. Cells were transiently transfected with 50 nM siRNA sequences using Lipofectamine 2000 (Invitrogen, United States). After 48–72 h of transfection, cells were harvested for further experiments.

### Western Blotting Assay

Western blot analysis was performed as previously described by us (Zhu et al., 2017). The following antibodies were used in the analysis: anti-SNAIL (Cell Signaling Technology, United States, lot:3895S) (at a 1:1,000 dilution), anti-MMP2 (Cell Signaling Technology, United States, lot:40994) (at a 1:1,000 dilution) and anti-β-actin (Cell Signaling Technology, United States, lot:3700) (at a 1:1,000 dilution); anti-N-cadherin (BD Biosciences, United States, lot:610920) (at a 1:1,000 dilution); anti-mouse (Cell Signaling Technology, United States, lot:91186S) (at a 1:2,000 dilution) and anti-rabbit (Cell Signaling Technology, United States, lot:98164S) (at a 1:2,000 dilution) secondary antibodies.

## Results

### Screening Differentially Expressed lncRNAs

**FIGURE 1:**
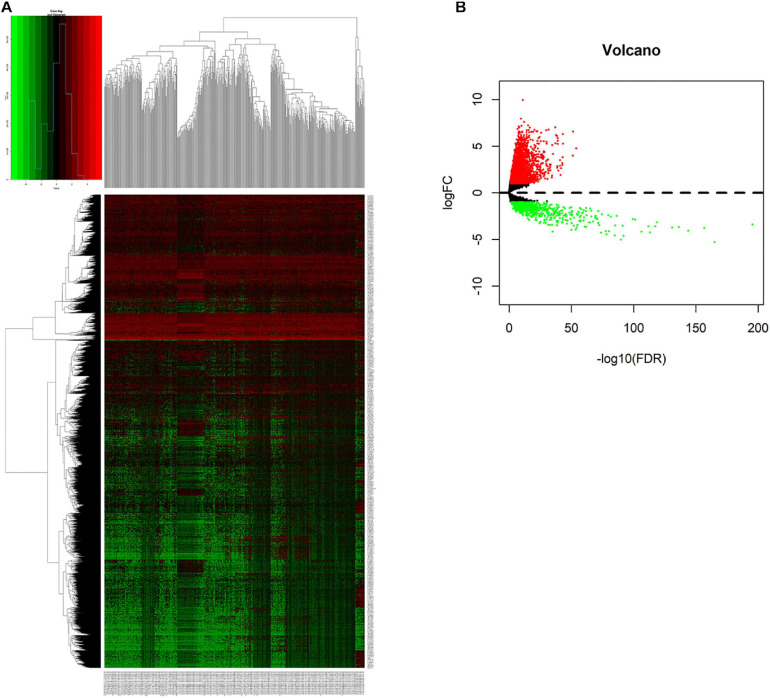
Screening differentially expressed lncRNAs. **(A)** Heatmap of differentially expressed lncRNAs in lung cancer from TCGA-LUAD dataset. Red and green indicate up-regulated and down-regulated lncRNAs respectively. **(B)** Volcano plot of differentially expressed lncRNAs in lung cancer from TCGA-LUAD dataset. Red and green indicate up-regulated and down-regulated lncRNAs respectively.

We compared lncRNAs expression profiles of the 497 LUAD samples with those of 54 normal samples. Then, we screened out 3,012 up-regulated lncRNAs and 761 down-regulated lncRNAs with a |fold change| > 1 and adjust *P* < 0.05. The heatmap and the volcano plot of the differentially expressed lncRNAs were visualized with the “ggplot2” and “pheatmap” packages of R software, and were shown in Figures 1A,B and Supplementary Figure 1.

### Construction of the Prognostic Risk Score Model

**FIGURE 2:**
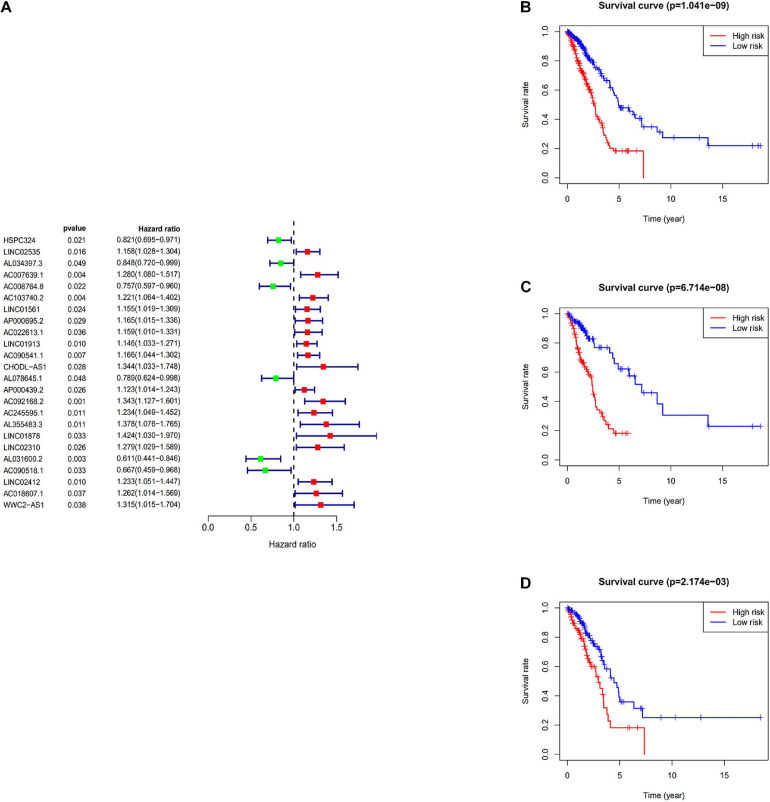
Construction of the prognostic risk score model. **(A)** Forest plot of univariate Cox regression analysis results of Immune-related lncRNAs. Red and green indicate risk and protective factors, respectively. **(B)** K–M survival curve of the immune-related lncRNA signature in the TCGA dataset. **(C)** K–M survival curve of the immune-related lncRNA signature in the training set. **(D)** K–M survival curve of the immune-related lncRNA signature in the testing set.

The immune-related lncRNAs were obtained by using the correlation test of immune-related genes (Pearson correction coefficient > 0.5, *P* < 0.05). A total of 24 immune-related lncRNAs were significantly correlated with the survival for TCGA-LUAD through the univariate Cox regression analysis (*P* < 0.05) (Figure 2A). All samples from the TCGA-LUAD dataset were randomly separated into the training set and the testing set. Then, all these 24 identified prognostic immune-related lncRNAs were analyzed with the multivariate Cox regression analysis (*P* < 0.05) in the training set; 10-lncRNAs with non-zero coefficients (LINC02535, AL034397.3, AC007639.1, CHODL-AS1, AL078645.1, LINC01878, AL031600.2, AC090518.1, LINC02412, and AC018607.1) were determined. Ultimately, 10 immune-related lncRNAs risk model was established, and the risk score of each patient was calculated using the following formula as a measure of survival risk:

LINC02535×0.14+AL034397.3×-0.16+AC007639.1×0.24+CHODL-AS1×0.22+AL078645.1×-0.22+LINC01878×0.70+AL031600.2×-0.55+AC090518.1×-0.64+LINC02412×0.17+AC018607⁢.1×0.43.

Based on the median risk score, samples were divided into the high-risk group and the low-risk group. In the training set, the KM survival curves showed that the model could distinguish the prognosis of patients well (Figure 2C). Similar results were also verified in the TCGA-LUAD dataset and the testing set (Figures 2B,D).

### Time-ROC Curve Analysis of Risk Model

**FIGURE 3:**
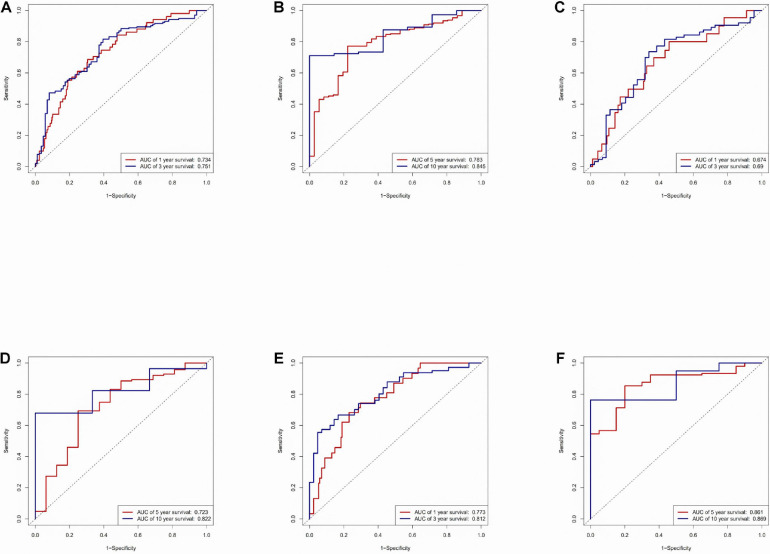
Time-ROC curve analysis of risk model. **(A)** Time-ROC curve analysis of the immune-related lncRNA signature in the TCGA dataset in 1, 3-year. **(B)** Time-ROC curve analysis of the immune-related lncRNA signature in the TCGA dataset in 5, 10-year. **(C)** Time-ROC curve analysis of the immune-related lncRNA signature in the testing set in 1, 3-year. **(D)** Time-ROC curve analysis of the immune-related lncRNA signature in the testing set in 5, 10-year. **(E)** Time-ROC curve analysis of the immune-related lncRNA signature in the training set in 1, 3-year. **(F)** Time-ROC curve analysis of the immune-related lncRNA signature in the training set in 5, 10-year.

An area under the ROC (AUC) curve was used to evaluate accuracy in the TCGA dataset, testing set, and training set. The area under the ROC (AUC) curve was applied to evaluate the accuracy of the prediction of the risk score model in 1-, 3-, 5-, and 10-year. The value of AUC indicates the model to predict well in prognostic prediction (Figure 3).

### Independent Prognostic Factor Analysis of the Risk Score Model With Clinical Risk Factors

**FIGURE 4:**
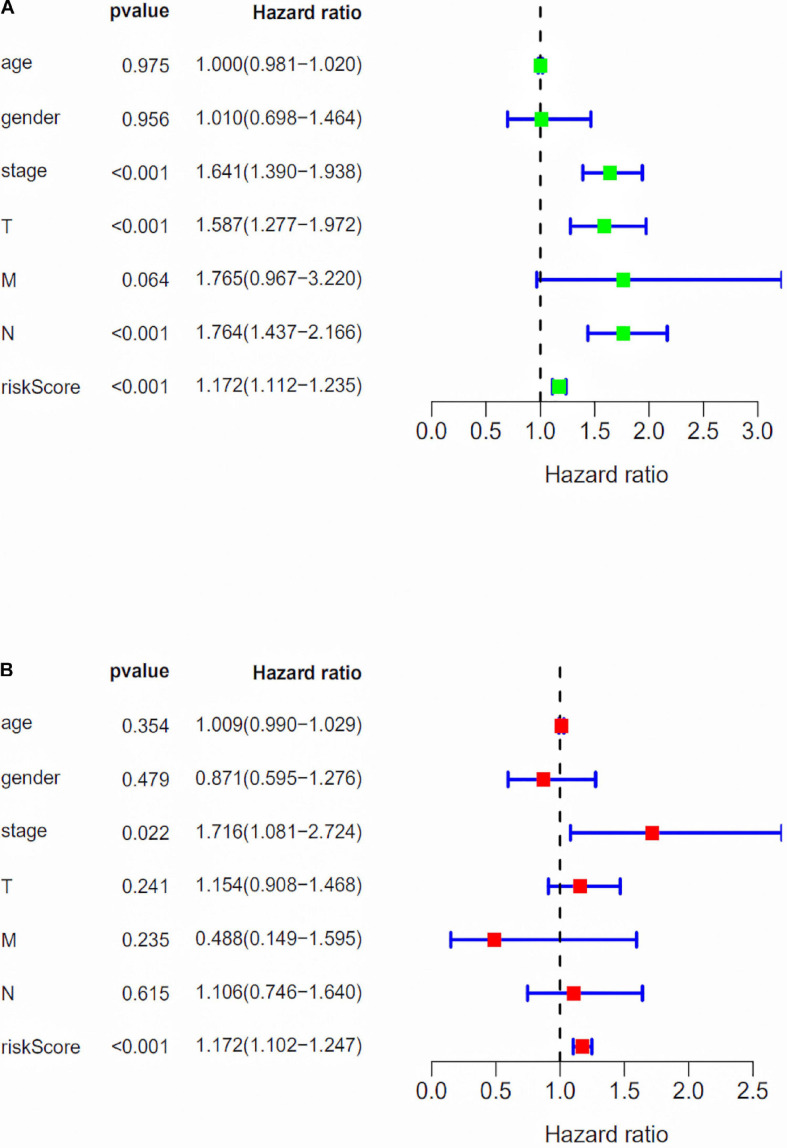
Independent prognostic factor analysis of the risk score model with clinical risk factors. **(A)** Forest plot of univariate Cox regression analysis. **(B)** Forest plot of multivariate Cox regression analysis.

We assessed the association between the risk score model and clinical risk factors such as the TNM stage, age, and gender by using univariate and multivariate Cox regression analyses. We found that the 10-lncRNAs signature was an independent predictor (Figure 4).

### GO Enrichment Analysis and KEGG Enrichment Analysis for the Differentially Expressed Genes Between the High-Risk Group and the Low-Risk Group

**FIGURE 5:**
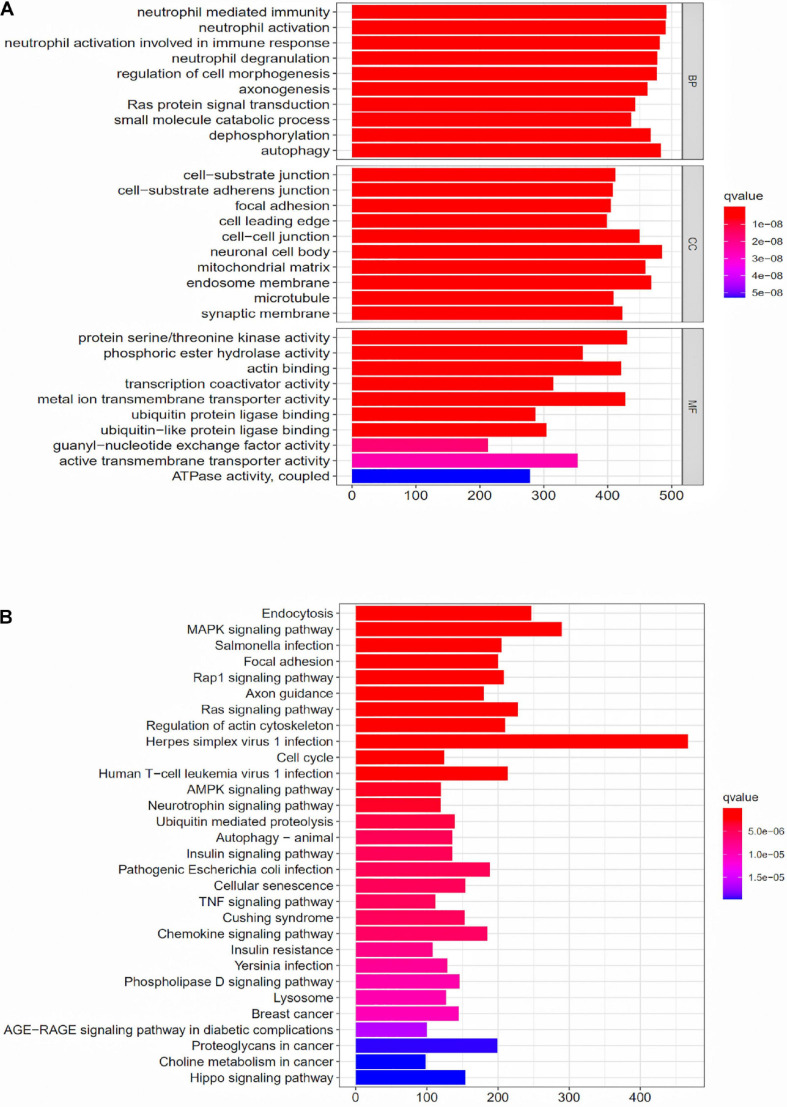
GO enrichment analysis and KEGG enrichment analysis for the differentially expressed genes between the high-risk group and the low-risk group. **(A)** GO enrichment analysis for the differentially expressed genes between the high-risk group and the low-risk group. **(B)** KEGG enrichment analysis for the differentially expressed genes between the high-risk group and the low-risk groups.

To further explore the potential function of the prognostic model, differentially expressed mRNAs analysis was performed between the high-risk group and the low-risk group. The most significant GO terms for biological process (BP), cellular component (CC), and molecular function (MF), as well as KEGG pathways, were analyzed to reveal potential biological functions of the differentially expressed mRNAs. These GO terms were primarily enriched in neutrophil mediated immunity, cell-substrate junction and protein serine/threonine kinase activity (Figure 5A). The KEGG indicated that the immune-related lncRNA signature was mainly enriched in Endocytosis, MAPK signaling pathway, Salmonella infection and Focal adhesion (Figure 5B).

### Analyzing Differentially Expressed Genes Related to the Immune Response Between the High-Risk Group and the Low-Risk Group

**FIGURE 6:**
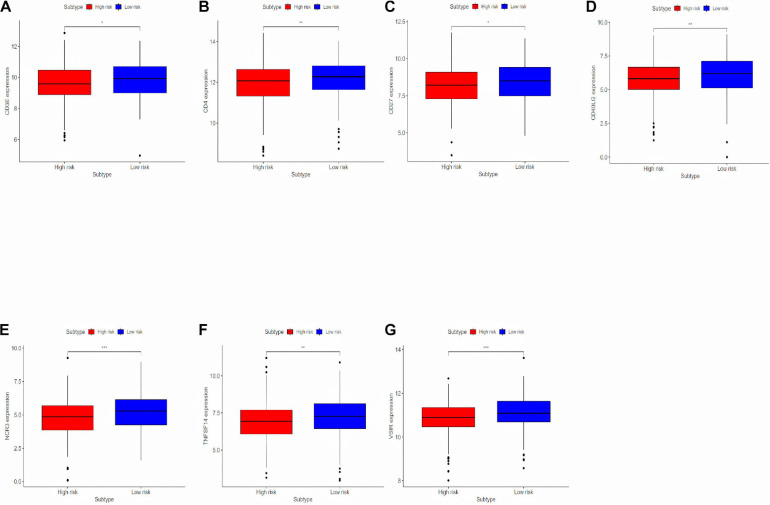
Analyzing differentially expressed genes related to the immune response between the high-risk group and the low-risk group. **(A–G)** Differentially expressed genes related to the immune response between the high-risk group and the low-risk group. *P < 0.05, **P < 0.01, and ***P < 0.001.

To deeply research the potential biological mechanisms of the prognostic model and its efficacy in predicting the efficacy of immunotherapy, differentially expressed immune response-related genes were analyzed between the high-risk and the low-risk group (Figure 6). The differences in immune-reactivity related genes between high-risk and low-risk groups are significant. The results showed that immune-reactivity related genes were highly expressed in the low-risk group.

### Analyzing Differentially Expressed Genes Related to the EMT Between the High-Risk Group and the Low-Risk Group

**FIGURE 7:**
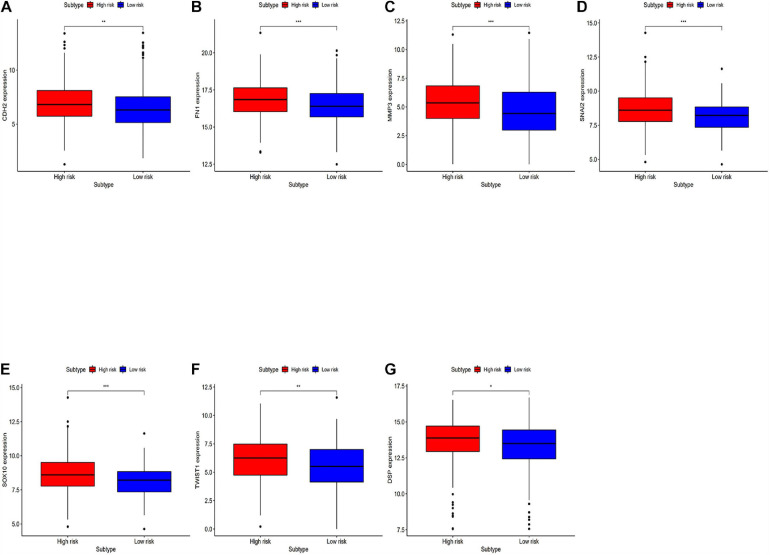
Analyzing differentially expressed genes related to EMT between the high-risk group and the low-risk groups. **(A–G)** Differentially expressed genes related to the immune response between the high-risk group and the low-risk group. *P < 0.05, **P < 0.01, and ***P < 0.001.

Differentially expressed EMT related genes between the high-risk and the low-risk were analyzed for deep-going exploration of the potential biological mechanisms of the prognostic model and its efficacy in predicting the efficacy of immunotherapy (Figure 7). The differences in EMT-related genes between high-risk and low-risk groups are significant. The results showed that EMT-related genes were lowly expressed in the low-risk group.

### Expression Level of 10 lncRNAs in Cell Lines as Detected by a qRT-PCR Assay

**FIGURE 8:**
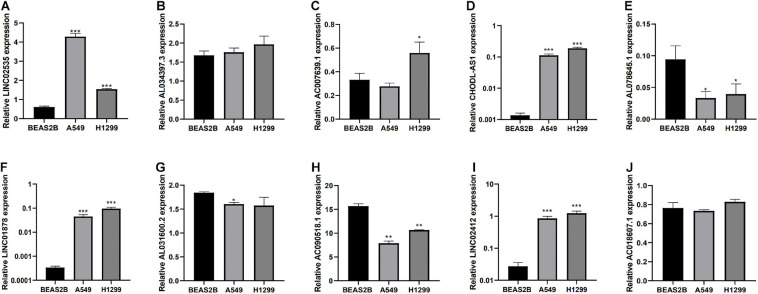
Expression of lncRNAs from the risk model in LUAD cell lines and bronchial epithelial cell. **(A–J)** Expression of 10 LncRNAs from the risk model in LUAD cancer cell lines and bronchial epithelial cell. *P < 0.05, **P < 0.01, and ***P < 0.001.

Finally, we detected the expression levels of 10 lncRNAs in BEAS2B, A549, and H1299 cell lines by a qRT-PCR assay. The results showed that LINC02535, AC007639.1, CHODL-AS1, LINC01878, and LINC01412 were highly expressed in LUAD cell lines, AL078645.1, AL031600.2, and AC090518.1 were lowly expressed in LUAD cell lines (Figure 8).

### Time-ROC Curve Analysis of Risk Model in the Independent Validation Dataset

**FIGURE 9:**
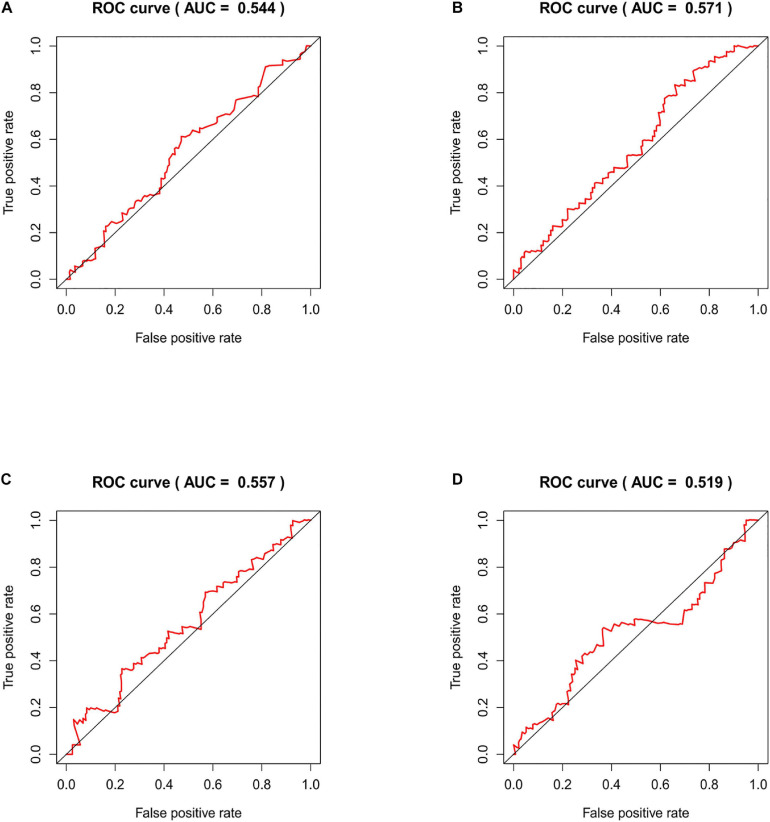
Time-ROC curve analysis of risk model in the independent validation dataset. **(A)** The AUC of ROC of 5 years about Immune-related lncRNA signature in the independent validation dataset. **(B)** The AUC of ROC of 5 years about Six-lncRNA signature (PMID 33324975) in the independent validation dataset. **(C)** The AUC of ROC of 5 years about seven-lncRNA signature (PMID 32596372) in the independent validation dataset. **(D)** The AUC of ROC of 5 years about seven-lncRNA signature (PMID 33163400) in the independent validation dataset.

We compared our risk model with other published prognostic signatures with data from PMID32015526 (Chen et al., 2020). The AUC of ROC of 5 years about Immune-related lncRNA signature was 0.544 in the independent validation dataset (Figure 9A). The AUC of ROC of 5 years about Six-lncRNA signature (PMID 33324975) was 0.571 in the independent validation dataset (Figure 9B). The AUC of ROC of 5 years about seven-lncRNA signature (PMID 32596372) was 0.557 in the independent validation dataset (Figure 9C). The AUC of ROC of 5 years about seven-lncRNA signature (PMID 33163400) was 0.519 in the independent validation dataset (Figure 9D). We find that the accuracy of the immune-related lncRNA signature prediction is as good as that of these models.

### RNA Interference and Western Blotting Assay

**FIGURE 10:**
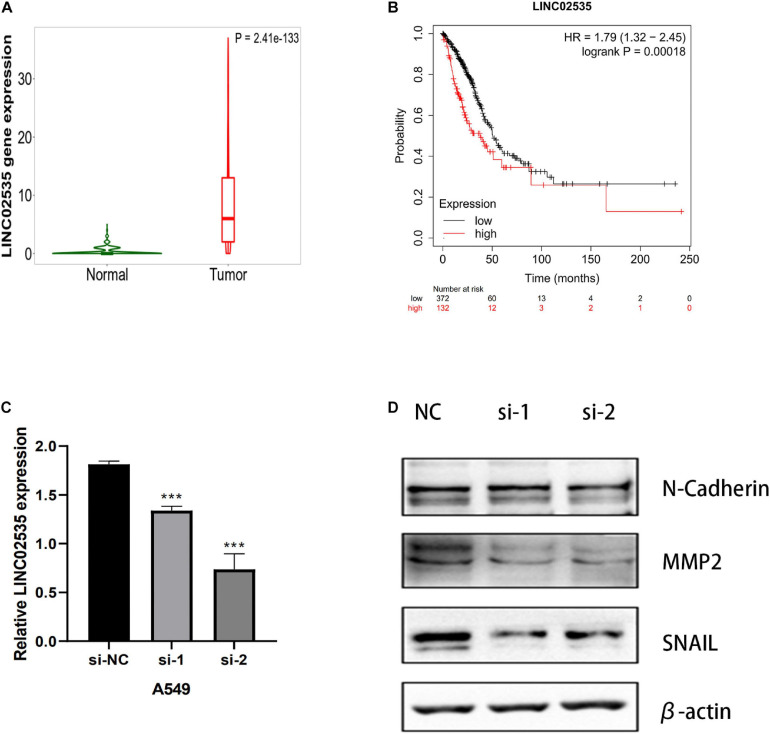
LINC02535 enhances the invasion and migration of LUAD cell *in vitro*. **(A)** The expression of LINC02535 in samples. **(B)** The survival analysis of LINC02535. **(C)** Quantitative real-time PCR analysis of LINC02535 expression in LINC02535-silenced cell and scrambled-siRNA-treated cell. **(D)** The protein levels of N-cadherin (CDH2, MMP2, and SNAIL were detected by Western blotting in the LINC02535-knockdown group. ***P < 0.001.

We next evaluated whether this risk model promoted the development of LUAD cells. After examining the fold-changes of the 10 immune-related lncRNAs, we selected LINC02535 for further functional assays. The EMT is a critical process during tumor invasion and metastasis. We measured the protein expression of EMT markers in siRNA-LINC02535-treated A549 cell. When cells were transfected with siRNA-LINC02535, the qRT-PCR analysis revealed that LINC02535 was significantly downregulated in the A549 cell after transfection (Figure 10C). The expression of SNAIL, MMP2, and N-Cadherin (CDH2) decreased in the A549 cell (Figure 10D). These results indicated that LINC02535 promoted the EMT and likely enhanced the migration and invasion of the A549 cell.

## Discussion

Nowadays, prognostic predictions for lung cancer patients largely rely on the American Joint Committee on Cancer TNM staging system. However, the TNM system is constrained by the assumption that there is a blunt correlation between anatomical disease progression and stage progression. Forcing patients into the same stage can introduce heterogeneity into clinical decision-making. Therefore, a reliable prognostic model for LUAD is urgently needed in the era of precision medicine.

In the present study, based on public high-throughput lncRNA expression profiles and clinical data from TCGA-LUAD Project, we discovered a novel 10-lncRNA signature that could effectively identify high-risk LUAD patients. These exhibited significantly shorter survival than those in the low-risk group.

The expression profile of immune-related lncRNAs in LUAD was identified by a correlation analysis of lncRNAs and immune-related genes. Ten immune-related lncRNAs were associated with OS (Overall Survival) in the training set by univariate Cox regression and multivariate Cox regression analyses. The 10 immune-related lncRNAs were used to construct a signature for the prediction of OS in LUAD patients in the training set (Figure 2). The risk scores generated from the expression levels of these 10 immune-related lncRNAs could accurately predict the OS of the patients in the set at 1, 3, 5, and 10 years (Figure 3). We assessed the association between the risk score model and clinical risk factors such as the TNM stage, age, and gender by using univariate and multivariate Cox regression analyses. We found that the 10-lncRNAs signature was an independent predictor (Figure 4).

Lung adenocarcinoma is a multifactorial disease with high heterogeneity and mixed genetic factors. Patients suffered from this disease tend to be diagnosed at an advanced stage and miss out on the chances of surgery. Despite improvements in lung cancer diagnosis, surgical techniques, and new drugs, lung cancer survival rates remain at an extremely low level. As we move into a new era of personalized medicine, it is becoming increasingly clear that many complex diseases, especially tumors, can rarely be attributed to a single genomic mutation. Therefore, it is important for us to conduct the genetic test for LUAD and to predict patient survival based on individual characteristics. Many experiments show that lncRNAs play a critical role in the maintenance of many biological processes. They are involved in the occurrence and development of LUAD and may be potential diagnostic and prognostic markers (He et al., 2020). In this study, to explore the prognostic value of lncRNAs in LUAD, we used the TCGA dataset to construct and identify the prognostic model. Finally, a stable 10-lncRNAs risk prognosis model was constructed. In the risk model, LINC02535 has been shown that can enhance the stability of the downstream gene RRM1 by combining PCBP2 in cervical cancer. It inhibits the repair of DNA damage in cervical cancer cells and furthers the process of EMT. Ultimately, it promotes the occurrence and development of cervical cancer (Wen et al., 2020). AL034397.3 was found to be correlated with autophagy in LUAD. However, the specific function and mechanism have not been reported (Zhou et al., 2020). DENG et al. found that the expression of CHOD1-AS1, in low-grade gliomas, was at the lower level. It may be a diagnostic and therapeutic target for low-grade gliomas, but it hasn’t been tested yet (Jianzhi Deng et al., 2019). LINC01878 may play a vital role in thyroid cancer, which also had no experimental confirmation (Zhang et al., 2019). In this model, the other lncRNAs have not been reported in the literature. It suggests that we can make further exploration. In addition, we demonstrated that risk score can be an independent predictor of LUAD by evaluating the clinical data and the risk model. Functional enrichment analysis revealed that these lncRNAs play significant roles in the development and progression of LUAD. GO enrichment analysis indicated that multiple biological functional differences exist between high-risk and low-risk groups (Figure 5A). Immunotherapy has been recognized worldwide in the treatment of NSCLC, the response rate in patients with high expression of PD-L1 can even reach about 30% and the median survival time has been extended to about 20 months (Mazieres et al., 2021). However, with the further application of immunotherapy, the researchers gradually found that even people with high expression of PD-L1 could not be fully benefited. Researchers from France published a retrospective study on JAMA Oncology looking for new ways to predict the effectiveness of immunotherapy. The study got the conclusion that patients with dNLR [Neutrophil number/(White blood cell number – Neutrophil number)] < 3 and Lactate dehydrogenase in the normal range, their relapse-free survival (RFS), and total survival were significantly prolonged after receiving immunotherapy (Takada et al., 2019). The inflammatory response has been shown to be connected with immune resistance in tumor patients, it can promote tumor proliferation and metastasis and activate multiple tumor signaling pathways (Hanahan and Weinberg, 2011). It suggests that our risk model is likely to predict patient immunotherapy outcomes. KEGG enrichment analysis indicated that there are multiple different signaling pathways between high-risk and low-risk groups (Figure 5B). MAPK is a widespread serine and threonine protein kinase in cells. It plays a key role in various signal transduction processes between mammalian cells. MAPK is mainly stimulated and activated by mitogen, cytokines, and neurotransmitters. MAPK transforms extracellular signals into intracellular signals and exerts biological effects by regulating the expression and function of relevant genes and proteins (Wang et al., 2017). In recent years, many researchers suggest that the MAPK signaling pathway may be in relationship with the formation process of tumor drug resistance (Chang et al., 2017). RBM10 was found to inhibit the cell proliferation of LUAD by inhibiting the Rap1/AKT/CREB signaling pathway (Jin et al., 2019). The French scientists found that RAS mutation is involved in regulating the expression of PD-L1 and directly affecting the immune response. In tumor cells, tristetraprolin protein (TTP) is responsible for the degradation of PD-L1 mRNA, which controls the expression of PD-L1. On the contrary, in tumor cells with RAS mutation, the RAS signaling pathway inhibits TTP activity, resulting in the non-degradation of PD-L1 mRNA and the stability of PD-L1 mRNA is increased. RAS gene mutation is expected to be a new marker for the prediction of the therapeutic effect of PD-1/PD-L1 antibody drugs (Coelho et al., 2017). AMPK is an AMP-dependent protein kinase and a key molecule in the regulation of energy metabolism. Activation of AMPK shuts down the anabolic pathway that consumes ATP and activates the catabolic pathway that produces ATP. Reuben Shaw et al. have found that advanced cancer can trigger cell recycling signals of AMPK, engulf cell debris, and provide nutrients needed for tumor growth. Blocking AMPK can prevent the most common advanced lung cancer from growing (Eichner et al., 2019). Yamamoto et al. (2020) found that MHC-I reduces antigen presentation by binding to autophagy receptor NBR1 and being transported to the lysosome for degradation, leading to immune escape of pancreatic cancer (PDAC). When autophagy is inhibited in PDAC, MHC-I in PDAC cells can be restored; furthermore, antigen presentation and anti-tumor immunity of CD8 + T cells are improved (Yamamoto et al., 2020). TNF inhibitors combined with CTLA-4 and PD-1 immunotherapy can improve the course of colitis in mice and improve the anti-tumor effect (Perez-Ruiz et al., 2019). The hippo pathway is an evolutionarily conserved signaling pathway that regulates organ size development, regeneration, and cancer in multicellular organisms. The Hippo pathway also plays an important role in the body’s immune function (Zhang et al., 2018). To explore whether the prognostic model can predict the immunotherapy response, we compared the differences in immune-reactivity related genes between high-risk and low-risk groups. We found remarkable differences in CD3E, CD4, CD27, CD40LG, NCR3, TNFSF14, and VSIR (Figures 6A–G; Zhang et al., 2020). All of these genes are recognized as marker genes closely related to the efficacy of immunotherapy. CD3E is a type I membrane glycoprotein on the surface of T cells, which is closely related to the T cell antigen receptor (TCR) on the lymphocyte surface. CD3E is reported to be involved in signal transduction within T cells after antigen recognition (Wang et al., 2019). CD3E and CD274, CD3D, and CD3G are both T-cell receptor genes and may be associated with tumor epigenetics (Bacolod et al., 2019). CD4, CD3, and CD8 are both surface markers of T cells, which are concerned with immunotherapy in tumors (Noda et al., 2020). CD27 is a co-stimulating immune checkpoint receptor expressed in T cells, NK cells, and B cells (Burchill et al., 2015). It binds to its ligand CD70 to regulate signal transduction. CD27 can promote the activation, proliferation and survival of T cells by upregulating Bcl-2 family members. CD27 enhances the anti-tumor effect of T cells responding to tumor antigens. CD40LG is a CD40 ligand, which has been reported to be relevant to LUAD and may be a protective factor (Xu et al., 2018). NCR3 is a natural killer cell receptor. Low NCR3 expression is associated with poor OS and progression-free survival (PFS) (Fend et al., 2017). TNFSF14 is a co-stimulating molecule that can regulate T cell activation. If it is forced expression in tumor cells, it can promote lymphoid structure formation to guide T cell aggregation and activation, resulting in tumor regression (Tang et al., 2016). VSIR belongs to the immune checkpoint family, also known as B7-H5. VSIR is considered as an important immunomodulatory factor in NSCLC, and its expression level is closely related to PD-L1 (Villarroel-Espindola et al., 2018). Gene signatures of immune cells correlate highly with EMT marker expression in tumors. In the pan-cancer analysis, several EMT-related genes can be significantly associated with worse patient outcomes (Gibbons and Creighton, 2018). The epithelial related gene is DSP. The mesenchymal related genes are CDH2, FN1, MMP3, SNAI2, SOX10, and TWIST1. PD-L1 is regulated by signaling pathways, transcription factors and epigenetic factors, such as EMT (Tuo et al., 2019). EMT is a crucial step in lung cancer progression, involving several morphological and phenotypical changes. CDH2 (N-cadherin) enhances migration and invasiveness (Marchetti et al., 2021). Low expression of FN1 is associated with long survival time in diffuse, poorly differentiated, and lymph node-positive gastric cancer. FN1 expression is associated with OS in patients with gastric cancer. FN1 may serve as promising targets for gastric cancer treatment (Han et al., 2020). miR-515-3p directly regulates MMP3 expression by binding to the coding sequence. MMP3 promotes tumor metastasis and thus represents a promising prognostic biomarker and therapeutic strategy in esophageal squamous cell carcinoma (Hu et al., 2020). Rhabdomyosarcoma (RMS) is an aggressive pediatric malignancy of the muscle that includes fusion positive (FP)-RMS harboring PAX3/7-FOXO1 and fusion negative (FN)-RMS commonly with RAS pathway mutations. RMS express myogenic master transcription factors MYOD and MYOG yet are unable to terminally differentiate. SNAI2 is highly expressed in FN-RMS, is oncogenic, blocks myogenic differentiation, and promotes growth. MYOD activates SNAI2 transcription *via* super enhancers with striped 3D contact architecture (Pomella et al., 2021). *In vitro* functional studies demonstrate that the subtype switch caused by the loss of SOX10 is analogous to the proneural-mesenchymal transition observed in patients at the transcriptomics, epigenetic, and phenotypic levels. SOX10 repression in an *in vivo* syngeneic graft glioblastoma mouse model results in increased tumor invasion (Wu et al., 2020). TANAR could impede nonsense-mediated mRNA decay (NMD) of TWIST1 mRNA by direct interaction with TWIST1 5′UTR. A preclinical study using a *in vivo* mouse model with orthotopic xenografts of clear cell renal cell carcinoma cells further confirmed the *in vitro* data. These results illustrated that AR-mediated TANAR signals might play a crucial role in clear cell renal cell carcinoma VM formation and metastasis, and targeting this newly identified AR/TANAR/TWIST1 signaling may help in the development of a novel anti-angiogenesis therapy to better suppress the clear cell renal cell carcinoma progression (You et al., 2021). We analyzed differences in the expression of EMT-related genes between the two risk groups, and the results were consistent with expectations (Figure 7). We detected the expression levels of 10 lncRNAs in BEAS2B, A549, and H1299 cell lines by a qRT-PCR assay. The results showed that LINC02535, AC007639.1, CHODL-AS1, LINC01878, and LINC01412 were highly expressed in LUAD cell lines; AL078645.1, AL031600.2, and AC090518.1 were lowly expressed LUAD cell lines; and AL0343973 and AC018607.1 had no significant difference in cells, which was considered to be related to the lack of cell types (Figure 8). We compared our risk model with other published prognostic signatures with data from PMID32015526 (Chen et al., 2020). We find that the accuracy of the immune-related lncRNA signature prediction is as good as that of these models (Figure 9).

We chose LINC02535 for further functional assays. By the Kaplan–Meier-plotter database^3^. LINC02535 is highly expressed in LUAD tissues (Figure 10A) and closely related to the OS of LUAD (Figure 10B). Knocking down LINC02535 also significantly inhibited the EMT, a key contributor to tumor invasion and metastasis, by inducing the expression of SNAIL, MMP2, and CDH2 (N-cadherin) (Figure 10D). Therefore, silencing LINC02535 in the A549 cell may reduce tumor motility and invasiveness.

In this study, the immune-related lncRNAs prediction model was established by univariate and multivariate Cox regression analysis. In order to verify the efficiency of our model, we used a KM survival curve to illustrate the survival time of the two groups. Furthermore, we validated our prognostic signature in an independent cohort. The AUC of ROC of 5 years was 0.544 in the independent validation dataset, which showed that this model had superior accuracy. In our research, the prediction signature was not only connected with the immune response but also with the specific biological mechanisms. Our prognostic model was also more superior than the other prognostic signatures by comparison.

In summary, we identified and validated 10 LncRNAs associated with survival time in the LUAD cohort. This risk model has been further demonstrated to be an effective tool for risk stratification and individual prognosis assessment. It provides an important reference for individualized clinical treatment of LUAD patients.

## Data Availability Statement

Publicly available datasets were analyzed in this study. This datacan be found here: https://portal.gdc.cancer.gov/, https://www.gsea-msigdb.org/gsea/index.jsp, http://kmplot.com/analysis/index.php?p=background, and http://www.cbioportal.org/study/summary?id=luad_oncosg_2020.

## Author Contributions

JH and ZL designed the experiments. YL, RS, and AW analyzed the data and wrote the manuscript. JZha, JZho, WZ, and RZ provided helpful discussion and reviewed the manuscript. All authors reviewed the manuscript.

## Conflict of Interest

The authors declare that the research was conducted in the absence of any commercial or financial relationships that could be construed as a potential conflict of interest.

## Footnotes

1 https://portal.gdc.cancer.gov/

2 https://www.gsea-msigdb.org/gsea/index.jsp

3 http://kmplot.com/analysis/index.php?p=background

## Supplementary Material

Supplementary Figure 1 Heatmap of differentially expressed lncRNAs in lung cancer from TCGA-LUAD dataset. Red and green colors indicate up-regulated and down-regulated lncRNAs, respectively.
